# Flexible weighing of olfactory and vector information in the desert ant *Cataglyphis fortis*

**DOI:** 10.1098/rsbl.2013.0070

**Published:** 2013-06-23

**Authors:** Cornelia Buehlmann, Bill S. Hansson, Markus Knaden

**Affiliations:** Max Planck Institute for Chemical Ecology, Hans-Knoell Strasse 8, 07745 Jena, Germany

**Keywords:** *Cataglyphis*, path integration, odour plume, homing, foraging, navigation

## Abstract

Desert ants, *Cataglyphis fortis*, are equipped with remarkable skills that enable them to navigate efficiently. When travelling between the nest and a previously visited feeding site, they perform path integration (PI), but pinpoint the nest or feeder by following odour plumes. Homing ants respond to nest plumes only when the path integrator indicates that they are near home. This is crucial, as homing ants often pass through plumes emanating from foreign nests and do not discriminate between the plume of their own and that of a foreign nest, but should absolutely avoid entering a wrong nest. Their behaviour towards food odours differs greatly. Here, we show that in ants on the way to food, olfactory information outweighs PI information. Although PI guides ants back to a learned feeder, the ants respond to food odours independently of whether or not they are close to the learned feeding site. This ability is beneficial, as new food sources—unlike foreign nests—never pose a threat but enable ants to shorten distances travelled while foraging. While it has been shown that navigating *C. fortis* ants rely strongly on PI, we report here that the ants retained the necessary flexibility in the use of PI.

## 1. Introduction

Desert ants have a remarkable navigational toolkit [1,2]. Path integration (PI) is performed continuously and takes into account walking distance and direction; moreover, it provides ants with a homeward vector pointing back to the starting point of their journey that is the nest [3]. PI is essential on early foraging trips but is prone to cumulative errors [4]. Therefore, experienced ants complement this strategy with landmark navigation, i.e. the use of place-specific olfactory and visual cues [5,6]. Once homing ants have got close to the nest, they eventually follow the odour plume emanating from the nest in order to pinpoint the entrance accurately [7].

Ants use these navigational strategies not only for localizing the nest, but also for returning to a familiar feeding site. PI guides them towards the known feeder, which is eventually pinpointed by its odour plume ([8] and references therein). In contrast to homing ants that compute a homeward vector from a current location to their home, ants heading for a familiar feeder first have to retrieve the coordinates from the feeder as recorded on previous visits. However, as in homeward vectors, information about direction and distance are not only encoded [8], but also integrated [9] in foodward vectors. Hence, PI is involved in both foodward and homeward runs.

**Figure 1.**
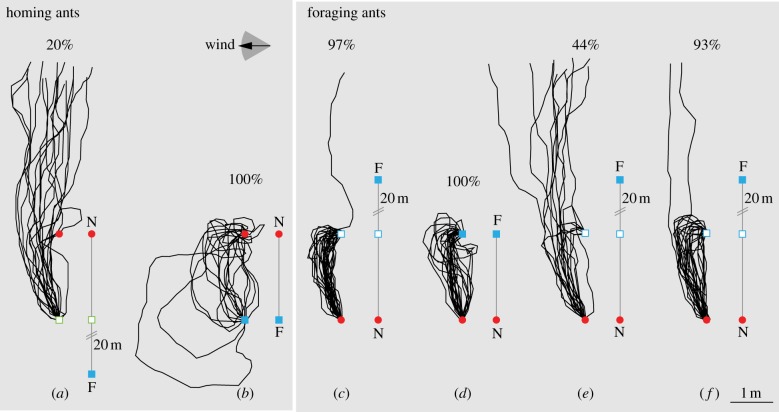
Olfactory and vector information in ants on the way home (*a*,*b*) and on the way to a familiar feeder (*c*–*f*). Ants were trained from the nest (red circle) to a feeder (filled blue square; nest-to-feeder distance, 20 m; except (*b*) and (*d*), 2-m control training paradigm). Homing ants (*a*) were captured at the feeder and released along the route 2 m away from the nest (green square). They still had a long PI vector when they encountered the nest plume and did not respond to the nest odour (*n* = 20 ants), whereas control ants (*b*) had run off their homeward vector when encountering the nest plume and directly followed the plume into the nest (*n* = 18 ants). (*a*,*b*) Adapted from Buehlmann *et al*. [7]. During tests with foraging ants on the way to the feeder, the odour source was placed 2 m away from the nest (empty blue square). Ants with a training distance of 20 m still had a long PI vector when they encountered the food plume ((*c*), *n* = 30 ants; (*e*), *n* = 18 ants; (*f*), *n* = 27 ants), but control ants in (*d*) had run off their foodward vector by the time they had reached the food odour (*n* = 25 ants). The feeder contained either cookie crumbs during both training and test (*a*–*d*), cookies during training but a dead cricket during test (*e*) or dead crickets during training and test (*f*). Numbers above trajectories depict the percentage of ants that followed the plume and neglected the PI vector. Raw data for the ant trajectories are stored at DRYAD (doi:10.5061/dryad.d1jk8).

In *Cataglyphis fortis,* it is known that PI is the predominant navigational strategy [1,2]. Furthermore, it was shown recently that homing ants are attracted to nest odours only when close to home, i.e. when their PI vector is run off (see figure 1*a,b* and also [7]). Here, we ask whether this dominance of vector over olfactory information is obligatory. Do ants on the way to a familiar feeding site only respond to the food odour when they are close to the expected feeder position?

## 2. Material and methods

### (a) Experimental site and ant species

Experiments were performed in the natural habitat of *C. fortis* during summer 2012 in a dry salt pan near the village of Menzel Chaker, Tunisia.

### (b) Experimental procedures

Ants were trained to a stable feeder (Petri dish containing cookie crumbs ad libitum) located 20 m away from the nest, with the nest-to-feeder direction being orthogonal to wind direction (figure 1*c*). In the 2-m control paradigm, both training and test distance of the feeder were 2 m from the nest (figure 1*d*). After at least half a day of training (approximately 15 foraging runs), we recorded the foraging trajectories of ants that left the nest heading for the learned feeder, while we placed identical cookie crumbs along the training route 2 m away from the nest (in the 2-m training paradigm there was no displacement of the feeder). Therefore, when encountering the food plume, test ants still had a long PI vector pointing to the feeder they had visited previously, while control ants had run off their vector completely. In an additional paradigm, we presented a small dead sun-dried cricket along the route to ants that were trained as before with cookies (figure 1*e*). In the final experiment, we trained and tested the ants with dead crickets (figure 1*f*). All ants were tested only once. Foraging runs were recorded on paper until the ants either reached the test feeder or overshot the feeder position for more than 4 m. A grid (mesh width, 1 m) on the ground served as a reference. The runs were digitized using Graph Click (v. 3.0). Only ants that passed the odour source downwind (i.e. on the side of the food-derived plume) were analysed and, as we do not know the functional reach of the different plumes, we analysed only those ants that crossed the plume within 1 m from the source. We conducted all experiments under similar wind conditions. The number of ants that pinpointed the source after contact with the odour plume was counted. Raw data for the ant trajectories are stored at DRYAD (doi:10.5061/dryad.d1jk8).

### (c) Statistics

Data were analysed using Fisher's exact test performed with the statistic software GraphPad Instat (v. 3.06). The *p*-values were adjusted by the Bonferroni correction.

## 3. Results

In a previous study [7], we showed that homing desert ants follow the nest plume only when they have run off the homeward vector (summarized in figure 1*a*,*b*; (*a*) versus (*b*), *p* < 0.05; see also [7]). Here, we test whether ants on the way to a familiar feeding site also do not respond to a food odour when they are still far away from the expected feeder location. Ants on the journey to a stable feeder (filled with cookie crumbs) 20 m away encountered the expected odour plume already along the route 18 m before the familiar feeder position was reached. Twenty-nine out of 30 ants followed the plume (figure 1*c*; (*c*) versus (*d*), *p* > 0.05). Because ants that have crossed the position upwind did not respond to the stimulus (see the electronic supplementary material, figure S1), we can exclude stimuli other than olfactory cues. Hence, unlike in homing ants, when they are on the way to food, olfactory input outweighs vector information.

Are ants at the start of a food vector primed to encounter the odour of a particular food? To approach this question, we trained ants as before but tested them with the odour of a single dead cricket by placing it at the test position described above. Here, only 10 out of 18 ants followed the plume, whereas the rest followed the PI vector to the feeder they had learnt (figure 1*e*; (*e*) versus (*c*,*d*), *p* < 0.05 each). But when ants were trained and tested with crickets 25 out of 27 ants followed the plume of a single cricket at 2 m from the nest (figure 1*f*; (*f*) versus (*c*), *p* > 0.05), indicating that the minute concentration present in the odour plume of a dead insect was sufficient for interruption of the journey, once the ants have experienced this odour before.

## 4. Discussion

We showed recently, not only that homing ants prefer to approach the nest from downwind in order to follow the odour plume, but also that ants follow the nest plume only when the path integrator tells them that they are close to home ([7]; summarized in figure 1*a*,*b*). Here, we provide evidence that this weighing of olfactory and vector information is not obligatory. Ants that are trained to a stable feeder pinpoint it using PI [8]. However, when they encounter a food plume, even though they may not have run off their foodward vector, they follow the food plume up to the food (figure 1*c*,*f*).

The difference between the responses to food and nest odours (figure 1*a*,*c*) could have been explained by the strong smell of cookies, which is probably more intense than the smell of a nest. However, this seems unlikely given that ants also responded to the odour of a single, small sun-dried cricket (figure 1*f*). We rather explain the different weighing in terms of functional benefits for the ants. Interestingly, ants followed the plume of a dead cricket more often when they had been trained to this kind of food (figure 1*e*,*f*). Hence, the interruption of a journey towards food is weaker if the food is common but has a different odour from what is expected, i.e. at the start of a foodward vector ants are primed to encounter the odour of a particular food. In wood ants and bees, it has been shown that the decision between choosing foodward or homeward journey is regulated by the animals’ feeding state [10,11]. We show that whether or not ants respond to a familiar odour plume depends not only on their behavioural state, being on the way to the nest or feeder, but also on the ants’ previous food-finding experiences and nutritional value of the food item (a dry cricket could have been less attractive in those experiments at that time than the cookies).

Our finding suggests that ants use PI and plume following as needed. The higher flexibility in ants returning to food compared with ants aiming for the nest is also found in the ants' searching pattern when the target is not encountered. Ants that do not encounter the nest after having run off the homeward vector centre their search on the expected nest location [12]. Although PI is involved in both homeward and foodward runs [9], the endpoint of a foodward journey is less strongly determined. When food is not encountered at the familiar position, the search strategy depends on their previous experience of food abundance and reliability [8]. Under natural conditions, without having experienced a plentiful feeder over a long time, ants tend to search beyond the expected food site [8,13].

As *C. fortis* does not discriminate the plume of its own nest from that of foreign ones, but encounters foreign nest plumes repeatedly when homing, it is essential for the survival to strictly follow the PI vector and respond to a nest plume only when the nest is almost reached and the value of the PI vector is close to zero. If not, the ant would risk following the wrong plume and being killed in a foreign nest. However, by responding to food plumes also when still far away from the food source they have learnt and still equipped with the foodward vector pointing at a learned food source, foraging ants retain the flexibility needed to adjust their behaviour and localize new food sources that might be situated closer to the nest. This allows the ants to reduce foraging distances as well as the time spent outside the nest that is beneficial in the harsh desert environment. One question which this study leaves open, but which will be answered elsewhere, is whether homing ants ignore all odours when their home vector is active or whether they ignore nest odours, but can still respond to food odours.

While it has been shown that *C. fortis* relies strongly on PI, we report here that the ants retained the necessary flexibility when performing PI by weighting vector and olfactory information depending of their behavioural state.
